# Alternative theories of COVID-19: social dimensions and information sources

**DOI:** 10.1057/s41271-025-00560-2

**Published:** 2025-02-19

**Authors:** Wesley Shrum, Paige Miller, Nana Osei Asiamah, Fangyue Zou

**Affiliations:** 1 Department of Sociology, Louisiana State University, 126 Stubbs Hall, Baton Rouge, LA 70803 USA; 2 Department of Sociology, University of Wisconsin, River Falls, WI USA

**Keywords:** Conspiracy, Vaccine, Information, Social media, Conservatism, Religiosity

## Abstract

While scientific understanding of the COVID-19 pandemic increased, conspiracy theories undermined the foundations of public health policy, making it significantly more difficult both to discuss and to implement. Popular alternative narratives include the claim that government was using restrictions to control people's behavior, and that the pandemic was caused by 5G cellular technology. We examine the extent to which alternative beliefs were associated with sociodemographic characteristics and the sources through which people acquired information during the pandemic. Our analysis uses a demographically balanced online survey of 10,022 participants from 50 US states, collected during August of 2021. Results indicate that those holding alternative theories tended to be right leaning, religious, young, male, and unvaccinated individuals. Sources of information were also strong predictors of such beliefs, specifically the extent to which social media were considered reliable.

**Supplementary Information:**

The online version contains supplementary material available at 10.1057/s41271-025-00560-2.

## Key messages

- Analyses showed beliefs in alternative theories of the COVID-19 pandemic tended to be stronger in respondents who were less educated, had lower income, were employed, male, right leaning, religious, and unvaccinated.
- Those who used social media and viewed it as reliable were more likely to support alternative beliefs.

## Introduction

By November of 2022, there were 519 million cases and 6.26 million deaths, related to the COVID-19 pandemic. Although scientific understanding of the pandemic has improved, alternative explanations regarding its origins and characteristics continue to hold sway among publics worldwide. Such explanations range from those with some scientific backing, like the ‘lab leak’ theory now supported by the FBI, to the more outlandish, like the notion that the virus was introduced and diffused for political gain. There are many explanations for the appeal of alternative theories and their ability to gain traction. The information landscape is complex, often contradictory [1] and social media provide a platform for the rapid spread of alternative theories [2, 3]. Such theories undermined public health policy during the main pandemic period by making public discussions more difficult to conduct and their outcomes more difficult to implement.

In this study, we aimed to assess the factors associated with belief in these alternative theories. We examined the extent to which alternative beliefs were associated with sociodemographic characteristics and the sources through which people acquired information during the pandemic. By identifying these factors, we provided insight into why such theories gained traction and why some people were more prone to belief, in the hope that its results can provide insights for policymakers.

## Data and methods

We collected data using an online survey during August of 2021. This period corresponds with the end of the pandemic wave with dominating Delta variant and the beginning of the Omicron-dominating wave.

The Alchemer software platform (https://www.alchemer.com) was used to invite respondents to complete an Internet-based survey. Eligible respondents within the Alchemer survey pool at the time of our survey numbered 25,098. These were US residents who have volunteered to take surveys in exchange for a small fee. Those who were over 18 years old and successfully completed the survey by August 30, 2021, were our respondents. A total of 10,022 cases from all 50 US states remained after eliminating incomplete surveys and disqualifying respondents who failed to correctly answer attention questions (e.g., check the color “red” below).

The dependent variable was a scale constructed by summing responses to seven alternative explanations for the pandemic. We applied summative scores that are conventionally used to enhance the reliability of construct measurement, reduce measurement error, and create more variability in response patterns. Respondents were asked to indicate whether they agreed that: (1) Coronavirus is a bioweapon developed by a government or terrorist organization, (2) Coronavirus was developed by pharmaceutical industries that will generate large profits, (3) Coronavirus was designed to reduce the world population, (4) China created the coronavirus in a laboratory for political gain, (5) Coronavirus is linked to 5G cellphone towers, (6) What has happened is all part a divine plan, and (7) The government is using coronavirus restrictions to see how much control they can exert over people's behavior. Summative scores were created by adding the respondent score for each of the seven variables. Level of agreement was indicated on a 4-point scale (from 1 = Strongly disagree to 4 = Strongly agree).

Independent variables in the study consist of demographic factors and questions about sources of COVID-19 information. Demographic dimensions include race (White, Black, Hispanic or Latino, Asian, and other), sex (male, female), age group (34 and younger, 35–64, and 65 and older), education level (dichotomized into those with a four year college degree or higher and those with less), income level (dichotomized into households earning above or below $50,000), employment status at the time of the survey, religiosity (frequency of religious service attendance), parental status (children/no children), and political identity (left, moderate, right). Vaccination status was indicated by a question on whether the respondent had received at least one dose of the COVID-19 vaccine by the time of the survey. We dichotomized the age variable at 55 years, since at the time our survey was fielded it had been widely publicized that older individuals were more at risk of serious illness or death.

Finally, we include eleven questions on information sources. Respondents were asked about the extent to which they used the following sources of information and the extent to which the sources were viewed as reliable: (1) local health experts, (2) national health experts (like the CDC), (3) World Health Organization, (4) work or school, (5) community or religious leaders, (6) family, (7) friends and acquaintances, (8) social media such as Facebook/Twitter, (9) local or regional government, (10) national government, and (11) broadcast, print, or online journalists. The frequency of using each source was measured on a 4-point scale (1 = frequently, 2 = sometimes, 3 = rarely, 4 = never). Source reliability was measured by asking “Thinking of the same sources of information, how reliable were they in helping you understand what was going on?” using a 4-point scale (1 = never, 2 = rarely, 3 = somewhat, 4 = very reliable). It should be noted that usage and reliability are extremely highly correlated and cannot be used in the same models and the results do not change no matter which one is used.

### Statistical analysis

We examined the extent to which alternative beliefs were associated with sociodemographic characteristics. We provided bivariate comparisons of summative scores based on dichotomized demographic characteristics. Due to the large sample size, differences of only 1.5% points are statistically significant at the 0.05 level. We also applied multiple linear regression to determine the relationship between the independent demographic variables (demographic and information resource variables) and the summative scores. The significance of all selected independent variables was tested in the preliminary regression model, resulting in a final model including only significant predictor variables. To ensure that the regression model meets the required assumptions, we conducted tests including (1) a link test to verify the relevance of the independent variables, (2) variance inflation factors that indicated multicollinearity was not an issue, and (3) minor homoscedasticity, corrected by using robust standard errors in the regression model.

Next, we examined the sources through which people acquired information during the pandemic using the described approach. We also conducted interaction analysis using the PROCESS macro version 4.2 for IBM SPSS Statistics 29. We estimated effects for individual demographic variables, then with all demographic factors, then with all demographic and information variables. We ran multiple models for all variables to determine whether the relationship between, for example, age and alternative beliefs differs with variation in political or religious views. However, the addition of interaction terms complicates the model without adding significant explanatory power.

## Results

We designed the sample to be demographically balanced in terms of self-reported region, race, and gender (Table 1). The distribution resembled the US population in terms of age (the bulk of the sample, 48.2%, were between the ages of 35 and 64; 24.6% 34 or younger, and 27.3% 65 or older) and education (equally divided into those who had and had not attended college). While our sample was selected to be demographically similar to the US, it was skewed slightly female (54%) with about equal percentages under 34 and over 65 years old. 80% of the sample is White, 12% Black, 6% Hispanic, and 4% Asian. About half were employed, earn over $50,000 annually, and have a college degree; 40% have children and about the same percentage attend religious services sometimes or frequently; 45% reported generally moderate political views, with 32% leaning right and 24% to the left. As of August 2021, 73% had at least one COVID vaccine.

**Table 1:** Sociodemographic characteristics

Variables	Frequency	Percentage
Ethnicity		
White	8065	80.5
Black	1212	12.1
Hispanic	568	5.7
Asian	397	4
Native American	243	2.4
Gender		
Female	5410	54
Children		
One or more children	3967	39.6
Age		
18–34	2470	24.6
35–64	4820	48.2
65+	2732	27.3
Education		
College or higher	4682	46.7
Income		
Over $50 K	5086	50.7
Vaccination status		
Had at least one COVID- vaccine	7262	72.5
Employment status		
Employed	4892	48.8
Religiosity		
Never	3173	32.5
Seldom	2530	25.9
Sometimes	1888	19.3
Frequently	2182	22.3
Political spectrum		
Left	2023	23.5
Moderate	3845	44.7
Right	2728	31.7

The extent of agreement with alternative theories and engagement with information sources, shown in Table 2, indicated that the majority do not agree with any of these statements. Most popular were theories related to coronavirus as government created either as a bioweapon (47%) or for political gain (48%). One widespread theory at the beginning of the pandemic linking the virus to 5G cellphone towers proved to be relatively unpopular (14%). Over one third (36%) agree that coronavirus was designed to reduce the world population. An even larger group (44%) believe that the government is using coronavirus restrictions to see how much control they can exert over people's behavior.

**Table 2:** Beliefs and information sources

Conspiracy beliefs^a^	% agree (*n*)	
Bioweapon	47.2 (4365)	
Profits	31.1 (2874)	
Population reduction	36.1 (3338)	
Laboratory	47.9 (4352)	
5G cell tower	13.9 (1241)	
Divine plan	33.2 (2929)	
Restrict behavior	44.1 (4180)	

Information sources^b^	% frequent use	% very reliable
National health experts (like CDC)	44.2 (4395)	37.8 (3701)
National government	31.7 (3130)	24.9 (2396)
Local health experts	31.5 (3114)	30.9 (2980)
World Health Organization	29.5 (2919)	32.2 (3064)
Family	26.4 (2579)	23.4 (2174)
Local or regional government	26.2 (2578)	22.2 (2123)
Broadcast, print or online journalist	25.1 (2458)	18.3 (1713)
Work or school	20.9 (1740)	17.7 (1311)
Friends and acquaintances	19.3 (1897)	16.9 (1561)
Social media such as Facebook Twitter	15.6 (1528)	10.8 (973)
Community or religious leaders	12.9 (1171)	16.3 (1275)

^a^Percentage reporting "agree strongly" or "agree somewhat"

^b^Percentage reporting "very reliable" and "frequently use source"

The second part of Table 2 describes how frequently respondents used various sources of information and their reliability. These two dimensions are highly correlated, since people tend to use the media, they view as reliable. National sources (such as governmental officials and health experts such as CDC) were used most frequently, followed by local health experts and the WHO. Family, local government, and journalists follow, used by about one quarter of the sample. Work and friends were used by about 20%, with social media and community/religious leaders used least often. Information sources were viewed as reliable in the same order, with exceptions at the high and low ends of the distribution. First, the percentage of respondents who viewed national sources as reliable is lower than the percentage who use them frequently. Second, social media such as Facebook and Twitter, used infrequently, were last in terms of perceived reliability, with only 10% viewing social media as very reliable. Two thirds of the sample viewed social media as non-reliable.

A bivariate analysis of the alternative belief scale and components is presented in Table 3. The first column shows average scores for demographic, behavioral, and attitudinal variables. The remaining columns show the percentage that agree ("strongly" or "somewhat") with each alterative theory. The first three comparisons show that socioeconomic factors (education, income, and employment) have relatively consistent relationships across alternative views. Those who are employed, with less education and income, are more likely to agree with all alternative theories apart from the relationship of COVID with 5G cell towers. Individuals with more education and income were more likely to hold this view, though the percentages are quite low.

**Table 3:** Bivariate analysis of beliefs and sociodemographic factors

	SCALE	Bioweapon^a^	Profits	Reduce pop	Lab virus	5G cell	Divine plan	Restrictions
Education^b^								
College or higher	13.75 (3748)	43.9% (1930)	27% (1201)	30.4% (1333)	44.3% (1928)	15.9% (691)	30% (1276)	39.8% (1800)
Less than college	14.44 (3539)	49.9% (2259)	34.1% (1537)	41% (1853)	51.2% (2267)	11.7% (500)	35.8% (1537)	47.7% (2204)
Income								
Greater than 50 K	13.97 (4029)	45.9% (2194)	28.6% (1375)	32.3% (1544)	47.6% (2245)	15.8% (740)	31.2% (1432)	42.2% (2060)
Less than 50 K	14.28 (3401)	48.5% (2118)	33.7% (1488)	40.1% (1758)	48.2% (2057)	11.8% (490)	35.2% (1548)	46.1% (2071)
Employed								
Yes	14.93 (3862)	48.8% (2221)	36.1% (1657)	39.8% (1824)	47.3% (2124)	20.7% (921)	37.6% (1662)	46.4% (2156)
No	13.17 (3467)	45.5% (2025)	25.2% (1119)	31.5% (1399)	48.3% (2110)	6.7% (287)	27.9% (1169)	41.5% (1902)
Gender								
Men	14.44 (3572)	50.7% (2182)	29.5% (1271)	34.7% (1480)	53.1% (2256)	14.8% (616)	31.1% (1276)	46.9% (2062)
Women	13.87 (3889)	44.4% (2170)	32.5% (1593)	37.4% (1847)	43.6% (2087)	13.3% (622)	35.2% (1649)	41.8% (2105)
Ethnicity^c^								
White	13.98 (6106)	47.7% (3562)	29.8% (2233)	34.6% (2591)	49.5% (3655)	12.3% (889)	31.0% (2217)	45.1% (3457)
Black	15.22 (829)	46.5% (504)	38.4% (412)	45.5% (491)	41.2% (426)	23.7% (243)	41.8% (492)	41.7% (457)
Hispanic	14.34 (436)	42.2% (226)	36.5% (194)	41.2% (219)	39.1% (202)	20.0% (100)	37.5% (188)	38.3% (207)
Asian	13.13 (307)	37.8% (138)	28.3% (102)	32.0% (116)	35.0% (125)	17.1% (60)	29.3% (105)	32.1% (119)
Native American	15.23 (163)	55.5% (122)	37.9% (85)	48.0% (106)	49.3% (107)	9.2% (19)	38.6% (80)	58.1% (133)
Age								
55 and over	12.75 (3326)	45.2% (1912)	20.5% (871)	26.3% (1111)	50.7% (2125)	3.5% (148)	24.0% (958)	40.6% (1778)
18–44	15.22 (4171)	49.0% (2453)	40.1% (2003)	44.3% (2227)	45.6% (2227)	22.7% (1093)	40.8% (1971)	47.2% (2402)
Children								
Yes	14.84 (4541)	52.0% (2911)	33.9% (1902)	39.4% (2208)	53.4% (2928)	15.8% (847)	36.2% (1941)	48.4% (2779)
No	13.03 (2956)	39.9% (1454)	26.7% (972)	31.0% (1130)	39.7% (1424)	11.2% (394)	18.5% (988)	37.6% (1401)
Attend religious services								
Frequent	16.27 (1666)	58.1% (1181)	37.2% (759)	41.3% (840)	60.4% (1222)	20.9% (407)	48.0% (937)	56.9% (1188)
Less than frequent	13.49 (5706)	44.0% (3077)	29.1% (2042)	34.3% (2401)	44.2% (3026)	11.9% (809)	28.8% (1923)	44.4% (2891)
Vaccinated								
Yes	12.96 (5624)	40.0% (2703)	23.4% (1582)	27.3% (1844)	42.7% (2835)	11.6% (772)	27.9% (1801)	34.5% (2384)
No	17.55 (1821)	66.8% (1602)	51.8% (1243)	59.7% (1441)	62.5% (1466)	20.3% (446)	47.1% (1077)	70.1% (1732)
Political Leaning								
Left	10.02 (1741)	17.1% (333)	11.6% (226)	14.2% (274)	16.6% (316)	6.1% (120)	15.4% (291)	13.9% (274)
Center	13.96 (2833)	44.2% (1553)	29.9% (1054)	34.1% (1201)	45.7% (1571)	14.8% (508)	33.0% (1105)	37.6% (1353)
Right	17.42 (2070)	70.8% (1828)	41.6% (1076)	48.5% (1242)	74.0% (1910)	20.1% (483)	43.7% (1072)	71.7% (1896)

All mean differences are significant at the .001 level

^a^Conspiracy variables were measured on a 4-point Likert scale where 1 = disagree strongly; 4 = agree strongly

^b^Differences of 1.5% or more are significant at the .05 level

^c^Ethnicity choices were not mutually exclusive, such that 5% of our sample selected more than one

Gender and ethnicity display varied relationships with alternative beliefs. While men score higher on the index (column 2), the relationship with gender is inconsistent for specific theories. Women are more likely to affiliate with the notion of pharmaceutical or divine origin, as well as population reduction, while men are more likely to agree the virus was created by the Chinese, as a bioweapon, or for government to exert control. Ethnicity is more difficult to interpret, because respondents were allowed to report more than one ethnicity. Indeed, over 5% of our respondents did so, such that direct comparisons between categories are not possible. However, in some cases, it is clear that different groups affiliate with different theories. Native Americans report the highest levels of agreement overall and with three of the alternatives (coronavirus as a bioweapon, designed to reduce the population, restrictions on people’s actions) while Black Americans report higher agreement with coronavirus as a tool of pharmaceutical companies, a divine plan, and cell towers. Whites were most likely to agree with the lab virus theory. Again, differences between groups were not large.

Age, the presence of children, and religiosity each had consistent relationships with alternative theories. Younger participants were more likely to accept alternative theories than older ones, with one exception. Older cohorts were more likely to agree the virus was created in a Chinese laboratory for political gain. Those with children and those who frequently attend religious services were also more likely to hold *all* of the alternative theories presented, with some differences as large as twenty percentage points.

While the size and unique contributions of these variables can only be observed using multivariate analysis, the magnitude of group differences is most apparent in the final rows of Table 3. For both the scale andthe specific alternative theories, unvaccinated individuals and those who are right leaning are more likely to express agreement. For three of these alternative views—coronavirus as a bioweapon, created by the Chinese for political gain, and used by the governments to restrict people’s actions—the difference between left- and right-leaning individuals is quite large, ranging from 54 to 58% points.

In Table 4, we use multiple regression to generate two models predicting belief in alternative theories. Independent variables in Model 1 include all sociodemographic variables as well as the perceived reliability of all eleven information sources. We note that if “reliability” is replaced with “usage,” the results remain the same. Seven of these sources are significant in predicting alternative beliefs, while four factors are not statistically significant (Work/School, Family, Local government, and Journalists). Model 2 contains only significant predictors and explains 41% of the variance in conspiracism.

**Table 4:** Multiple regression of beliefs on sociodemographic and information sources

	Model 1				Model 2			
	Unstandardized coefficients		Standardized coefficients	Sig	Unstandardized coefficients		Standardized coefficients	Sig
	B	Std. Error	Beta		B	Std. Error	Beta	
(Constant)	12.476	0.616		< .001	12.842	0.566		< .001
Income (50 K or higher)	− 0.502	0.181	− 0.038	0.006	− 0.506	0.164	− 0.039	0.002
Age	− 0.575	0.056	− 0.156	< .001	− 0.564	0.051	− 0.16	< .001
Education (college or higher)	− 0.368	0.173	− 0.029	0.034	− 0.35	0.158	− 0.027	0.027
Gender	− 0.447	0.164	− 0.035	0.006	− 0.425	0.15	− 0.034	0.004
Vaccinated	− 1.691	0.197	− 0.115	< .001	− 1.7	0.184	− 0.115	< .001
Currently employed	0.428	0.193	0.032	0.027	0.534	0.175	0.042	0.002
Children (one or more)	1.432	0.175	0.108	< .001	1.259	0.158	0.096	< .001
Religiosity (frequently attend religious services)	0.739	0.08	0.131	< .001	0.693	0.072	0.125	< .001
Political views (1 = left, 2 = moderate, 3 = right)	1.966	0.124	0.227	< .001	1.987	0.112	0.235	< .001
Reliability of information source								
Local health experts	− 0.323	0.134	− 0.043	0.016	− 0.355	0.116	− 0.048	0.002
National health experts (like the CDC)	− 0.875	0.146	− 0.135	< .001	− 0.983	0.134	− 0.153	< .001
World Health Organization	− 0.351	0.128	− 0.058	0.006	− 0.317	0.116	− 0.053	0.006
Work or school	− 0.085	0.118	− 0.012	0.475				
Community or religious leaders	0.818	0.113	0.128	< .001	0.727	0.095	0.113	< .001
Family	− 0.002	0.129	0	0.989				
Friends and acquaintances	0.467	0.138	0.06	< .001	0.442	0.108	0.056	< .001
Social media such as Facebook Twitter	1.421	0.103	0.233	< .001	1.339	0.091	0.219	< .001
Local or regional government	− 0.043	0.133	− 0.006	0.745				
National government	− 0.481	0.134	− 0.074	< .001	− 0.589	0.107	− 0.092	< .001
Broadcast, print or online journalists	− 0.137	0.12	− 0.021	0.256				
Adjusted *R*^2^	0.398				0.409			
Std. error of the estimate	4.97				4.87			

In the first part of the table, standardized coefficients suggest that alternative beliefs are negatively related to income, age, and education as well as gender and vaccination status, with women and vaccinated persons less likely to hold these beliefs. Such theories are positively related to current employment, having children, religiosity, and right-leaning political beliefs. Of this group of variables, the strongest predictors are age, vaccination status, and political/religious factors: younger, unvaccinated, right-leaning persons who attend church frequently are significantly more likely than their counterparts to agree with alternative theories. Perceived reliability is important as well. Those who gave high ratings to community leaders, friends, and social media also had higher conspiracy scores. Those who rated local and national health experts, WHO, and national government highly had fewer alternative beliefs. In terms of explained variance, the best predictors were low credibility ratings of national health experts (like CDC) and high ratings of community leaders and social media.

## Discussion

Our findings show that women, older individuals, and those with more education and income are less likely to believe in alternative theories. Those who are employed, more conservative, have children, and regularly attend religious services are more likely to believe such theories. In terms of social factors, the strongest associations are between vaccination status and political/religious dimensions with alternative beliefs. Indeed, position on the political spectrum is the single most powerful predictor of such beliefs. Other US studies have found that conservatives are more likely to affiliate with conspiracist thinking than liberals [4–8]. Early in the pandemic, this may have been in response to criticism of President Trump’s handling of the pandemic, which continued in the wake of the Presidential transition. International studies show that both left and right-wing ideologies may be associated with alternative beliefs, suggesting that future studies should contextualize the particular meanings of right and left, as well as the particular conspiracies at issue [9]. Religiosity, measured by frequent attendance at religious services, was also associated with alternative beliefs, a finding consistent with results from the US [8, 10]. That the unvaccinated are more likely to subscribe to these theories, even controlling for political views, is consistent with the notion that there is some causal connection. It may be that conspiracist beliefs reduce the motivation to vaccinate.

Demographically, alternative beliefs tend to be held by less educated, younger men with lower incomes. Age, one of the most powerful predictors, is negatively related to belief. Most of the literature would lead us to expect this including studies from Cyprus, Greece, Poland, England, Germany, Switzerland, and the United States [9]. Uscinski and colleagues attribute this finding to greater age-related experience in dealing with misinformation [8]. However, older people prove more conspiratorial in studies from Portugal, Brazil, and the United Kingdom [9, 11]. One widespread notion is that younger people consume more social media, where they are exposed to conspiracy theories [12, 13]. However, our models control for social media exposure, so the observed age effect here is independent of source.

Differences by SES are relatively small and may indicate different audiences for various alternative beliefs. Individuals with higher education and income were *less* likely to agree with most statements, but *more* likely to agree with the 5G theory. The finding that men are more likely than women to subscribe to alternative beliefs is consistent with many previous studies [14], including a meta-analysis of conspiracy beliefs [15]. However, other multinational samples find women more likely to adopt such beliefs [16]. Alternative beliefs about COVID are also more likely among employed persons, an association we find more difficult to explain. Perhaps employed persons spent more time discussing COVID with workmates online and encountered a greater range of COVID beliefs without opportunities to assess their validity. But many people had returned to the offices by the time of our survey, so the finding is also consistent with face-to-face facilitation.

Sources of information were also significant predictors of alternative beliefs. While we measured both frequency of use and perceived reliability of source, the two are highly correlated. Higher scores were associated with perceived reliability of community leaders, friends, and social media, while those who believed local and national health experts, WHO, and government experts had fewer alternative beliefs. The finding may be summarized by saying those who utilize and trust experts–whether local, national, or international–are unlikely to believe in alternative theories. Those who use and trust othe*r* sources–whether community leaders, friends, or social media–are more likely to affiliate with many alternative beliefs.

Trust in social media was the strongest predictor of belief in alternative theories. Commentators and scholars have often supposed that right-leaning posts, retweets, and misinformation on social media are a major factor in conspiracism [17, 18]. While our approach generally supports disentangling claims about conspiracy from claims about the accuracy of information, these findings support the idea that conservatism and social media have independent relationships with alternative beliefs. Prior studies have correlated trust and use of social media with conspiratorial thinking [19, 20]. However, our results show that the perceived reliability of social media is a stronger predictor than any other type of information. Social media was the second-best predictor overall, behind only political beliefs. Indeed, a regression model containing only these two variables explains 26% of the total variation the scale, as compared with 40% for one including all nine demographic and eleven information sources. This observation was supported by the recent Pew studies showing that about half of those under age 30 trust social media as much as national news sources [13].

This study has four limitations. First, inferences about causality are not warranted given the cross-sectional nature of the data. Second, ethnicity was not included as a predictor in the regression analysis because our question allowed for multiple choices. Simply distinguishing between white and non-white was not significant controlling for other factors. Third, the salience of specific conspiratorial ideas evolves over time. The conspiracy-related questions included here focused on those widely circulated before August 2021. However, there were other alternative theories about COVID-19 toward the end of the pandemic. Finally, research is needed to explore cross-cultural differences in the prevalence of alternative beliefs about COVID-19.

The seven examined statements expressed beliefs that were inconsistent with official accounts of the emergence of COVID-19. All but one of the statements involves some kind of disguised or hidden plot on the part of powerful actors [21]. One reason we utilize the notion of ‘alternative’ rather than ‘conspiracy’ theories was our sympathy with the critique that our ‘mid-pandemic’ survey was conducted during a period when knowledge was incomplete, and information was shifting. Because our survey items are all explanations that ran contrary to official accounts at the time, they preclude an analysis of the way in which official accounts, from government, medical officials, academics, and journalists may be conspiratorial. While it is beyond the scope of this paper to engage this critique, it is an important point. Second, our team of researchers includes both demographic and viewpoint diversity. The inclusion of the ‘divine plan’ idea is not at all conspiratorial for team members who believe that everything is part of a divine plan. We included this statement owing to its non-scientific origin, making it an ‘alternative’ account.

In the case of viral pathogens such as COVID-19, it is crucial that people have access to accurate information about the virus and its related vaccines. Misinformation about COVID-19 can lead to difficult if not conflictual policy discussions of public health as well as impede efforts to contain the pandemic. These explanations for the emergence of COVID-19 exist in a policy context where information evolves in real time. Early on, media reports highlighted the fact that the elderly and those with pre-existing conditions were most at risk of serious complications from the virus and that masking, particularly in closed quarters, and social distancing were effective means of slowing the spread. However, government agencies changed course on the effectiveness of masking, while some cities and local municipalities required masking even in public parks. In instances where medical experts or political leaders disagree among themselves or even appear to contradict themselves over time, transparency regarding uncertainties and decision-making processes is especially vital, particularly if they hope to reach those who distrust authority figures. This becomes even more pronounced in a context of waning trust in institutions of all types.

## Conclusions

Alternative theories tended to be supported by those who are younger, male, with less education, and lower incomes. They are also supported by those who are employed, right leaning, religious, unvaccinated, and who view social media as reliable. It is clear from this and other studies that alternative theories have a negative impact on public health [11]. This phenomenon is not unique to COVID-19, as similar patterns have been observed in other health crises and events. Understanding why people hold these beliefs and what factors contribute to their persistence is crucial in combating misinformation. Education and exposure to reliable sources of information are critical in promoting scientific literacy and combating the spread of alternative theories. Efforts must be made by governments, public health organizations, and the media to provide accurate information and to counteract the spread of false information.

## Supplementary Information

Below is the link to the electronic supplementary material.

Supplementary file1 (DOCX 18 kb)

## Data Availability

The data described in this Brief will be available at the Open Science Framework, Wisconsin COVID Project, following the publication of the final report.
