# Supplementary material for: Alternative theories of COVID-19: social dimensions and information sources
Source: J Public Health Policy. 2025 Feb 19;46(2):444–59. doi: 10.1057/s41271-025-00560-2 (PMC12119318; doi:10.1057/s41271-025-00560-2)
Supplement: Supplementary file 1 — Supplementary file1 (DOCX 18 kb) [file 41271_2025_560_MOESM1_ESM.docx]

**Literature on Conspiracies and Alternative Theories**

**Why People Believe?**

The literature on this subject includes a body of work on why people are drawn to alternative theories as well as social dimensions correlated with belief. While the nature of the theories themselves is beyond the scope of our analysis, many incorporate three elements. First, “conspiracies” attempt to explain major historical or contemporary events by suggesting a small but powerful group of interconnected individuals plot to bring about some nefarious end, one that often serves to further secure their power. Second, theories often assume a coverup on the part of those conspiring. Third, the outcomes of events are often detrimental to the common good. Frequently derided as irrational and popular primarily among those living at the margins of mainstream social life, many non-mainstream theories are widely held.

Locke re-crafts Max Weber’s 1904 rationalization thesis as an analysis of the developmental logic of theories of suffering, arguing that conspiracy culture is an outcome of moral accounting, or blame attribution, that informs mundane reasoning in modernity [1]. Rilinger shifts the focus from conspiracists to audience and proposes a relational theory of complex secrets [2]. He argues that corporate crimes often remain secret even after crucial information has become public, owing to bureaucratic hierarchies that allow criminal activity to be hidden. The author suggests that the uncovering of such complex secrets requires discovery and assembly of sets of information relative to a guiding conception of the whole. Sunstein and Vermeule argue that conspiracy theories, particularly those related to terrorism post-9/11, stem from a crippled epistemology caused by limited access to relevant information sources [3]. They argue the acceptance of alternative theories by individuals within isolated groups is not irrational, but merely unjustified compared to the information available in the wider society. Pigden challenges the notion that such theories are synonymous with superstition and argues that conspiracy is a normal part of political life [4]. He cites the works of historical figures such as David Hume to demonstrate that even highly sophisticated individuals have posited alternative theories.

In their review of psychological research on conspiracy theories, Douglas and colleagues find that belief is driven by epistemic motives related to understanding the environment, existential feelings of safety, and maintaining a positive self-image [5]. Others, such as Van Prooijen, examine the role of societal crises in adherence to alternative theories [6]. They argue that such situations, characterized by rapid change and distrust of established power structures, stimulate belief through feelings of fear, uncertainty, and a lack of control. Conspiracies become coherent historical narratives that may be transmitted to future generations and become part of collective mental representations of important historical events despite lack of evidence. It is worth noting that alternative theories can encompass a wide range of topics and events. For example, individuals who tend to believe in alternative theories are more likely to be skeptical of official explanations of events such as the September 11th terrorist attacks or the assassination of President John F. Kennedy [7].

**Who Believes?**

Harambam and Aupers conducted an ethnographic study of conspiracy culture, identifying three distinct types of identities [8]. They found many individuals actively resist stigmatization as ‘conspiracy theorists’ by distinguishing themselves as critical freethinkers. The authors conclude that this culture is not monolithic, but rather a network of different groups of people, identifying with different worldviews, beliefs, and practices. The primary identities are as activists, retreaters, and mediators. Activists engage in various forms of political activism, whereas retreaters believe in transforming the self and finding the good within. Mediators share the critique of activism and resistance with retreaters but believe in reaching out to inform and influence the general public.

Recently, Van der Linden and colleagues focused on the relationship between ideology and conspiratorial thinking in the United States [9]. The authors find that conservatives are more likely to embrace conspiratorial thinking than liberals and that this relationship is mediated by distrust of officialdom and paranoid ideation, characteristics that are more pronounced among conservatives. This finding aligns with the historical, philosophical, and scientific reasons outlined by Hofstadter's famous essay, “The Paranoid Style in American Politics” [10]. Stasielowicz employed meta-analysis, finding that several personality traits, including greater religiosity and cynicism, lower levels of political trust, and lower levels of cognitive ability were consistently associated with belief in conspiracy theories [11].

The importance of such beliefs resides in their policy consequences. During the pandemic those with a commitment to alternative explanations and conspiracies were found to be less likely to wear masks, engage in social distancing, and receive the COVID-19 vaccine [12]. Belief in conspiracy theories can also lead to increased distrust in established social systems, lower support for government policy and directives, and lower levels of social engagement more generally [13].

**References**

1. Locke S. Conspiracy culture, blame culture, and rationalisation. Soc Rev. 2009. 57:567-85.

2. Rilinger G. Corporate conspiracies and complex secrets: structure and perception of the insull scheme in 1930s Chicago. Am J Soc. 2019. 124:1043-89.

3. Sunstein CR, Vermeule CA. Conspiracy theories: causes and cures. J Pol Phil. 2009; 17:202-27.

4. Pigden C. Complots of mischief. In: Coady, D, editor. Conspiracy theories: The philosophical debate. Routledge. 2019. Pp. 139-66.

5. Douglas KM, Sutton RM, Cichocka A. The psychology of conspiracy theories. Cur Dir Psy Sci. 2017; 26:538-42.

6. Van Prooijen J-W, Douglas KM. Conspiracy theories as part of history: The role of societal crisis situations. Memory Studies 2017; 10:323-33.

7. Jolley D, Douglas, KM. The effects of conspiracy theories on attitudes, behavior, and psychological outcomes. Journal of Medical Internet Research. 2020; 22: e17866.

8. Harambam J, Aupers S. ‘I am not a conspiracy theorist’: Relational identifications in the Dutch conspiracy milieu. Cul Soc. 2017; 11:113-29. doi: 10.1177/1749975516661959.

9. Van der Linden S, et al. The paranoid style in American politics revisited: An ideological asymmetry in conspiratorial thinking. Political Psychology. 2021; 42:23-51.

10. Hofstadter R. The paranoid style in American politics. Harpers Magazine. 1964; November: 77-86.

11. Stasielowicz L. Who believes in conspiracy theories? J Res Personality. 2022; [doi:org/10.1016/j.jrp.2022.104229](https://doi.org/10.1016/j.jrp.2022.104229).

12. Bierwiaczonek K, Kunst JR, Pich O. Belief in Covid-19 conspiracy theories reduces social distancing over time. Applied Psychology: Health and Well-Being. 2020; 12:1270–85.

13. Pummerer L, et al. Conspiracy theories and their societal effects during the COVID-19 pandemic. Soc Psy and Pers Sci. 2022; 13: 49-59.1
